# Biosilica from Living Diatoms: Investigations on Biocompatibility of Bare and Chemically Modified *Thalassiosira weissflogii* Silica Shells

**DOI:** 10.3390/bioengineering3040035

**Published:** 2016-12-16

**Authors:** Stefania Roberta Cicco, Danilo Vona, Roberto Gristina, Eloisa Sardella, Roberta Ragni, Marco Lo Presti, Gianluca Maria Farinola

**Affiliations:** 1 Italian National Council for Research—Institute for the Chemistry of OrganoMetallic Compounds (CNR-ICCOM)-Bari, Bari 70126, Italy; cicco@ba.iccom.cnr.it; 2 Department of Chemistry, Università degli Studi di Bari Aldo Moro, Bari 70121, Italy; danilo.vona@uniba.it (D.V.); roberta.ragni@uniba.it (R.R.); marco.lopresti@uniba.it (M.L.P.); 3 CNR-Nanotech, Lecce 73100, Italy; roberto.gristina@cnr.it (R.G.); eloisa.sardella@cnr.it (E.S.)

**Keywords:** diatoms, biosilica, cell growth

## Abstract

In the past decade, mesoporous silica nanoparticles (MSNs) with a large surface area and pore volume have attracted considerable attention for their application in drug delivery and biomedicine. Here we propose biosilica from diatoms as an alternative source of mesoporous materials in the field of multifunctional supports for cell growth: the biosilica surfaces were chemically modified by traditional silanization methods resulting in diatom silica microparticles functionalized with 3-mercaptopropyl-trimethoxysilane (MPTMS) and 3-aminopropyl-triethoxysilane (APTES). Fourier transform infrared spectroscopy and X-ray photoelectron spectroscopy analyses revealed that the –SH or –NH_2_ were successfully grafted onto the biosilica surface. The relationship among the type of functional groups and the cell viability was established as well as the interaction of the cells with the nanoporosity of frustules. These results show that diatom microparticles are promising natural biomaterials suitable for cell growth, and that the surfaces, owing to the mercapto groups, exhibit good biocompatibility.

## 1. Introduction

Mesoporous silica nanoparticles (MSNs) are considered to be the most promising candidates for designing highly robust and tunable substrates for biomedical applications. Their biocompatibility, the high surface area-to-volume ratio, the possibility of an easy introduction of various organic functional groups (either through covalent bonding or electrostatic interactions), provide a remarkable level of versatility for these materials [1,2,3,4,5].

However, the synthesis of the MSNs is often time-consuming, expensive and complicated, involving toxic materials. Nature, on the other hand, has developed elegant self-assembly-based synthetic routes to produce biosilica with complex 3-dimensional (3D) porous structures [6].

The most outstanding example are diatoms, single-cell photosynthetic algae, with distinct silica cell walls called frustules, consisting of highly ordered pore structures, species characteristic patterns and hierarchical pore organization with unique mechanical, molecular transport, optical and photonic properties [7,8]. The biosilica of diatoms has currently found attractive applications in optics [9], photonics [10], sensing [11], biosensing [12], filtration [13], microfabrications [14,15], protein separation [16], catalyses [17] and drug delivery [18,19].

The potential of diatom biosilica for nanotechnological purposes lies in the structure of the frustules. Moreover, the free hydroxyl groups on the surface have been used for chemical modification of the surface and subsequent tethering of biological or chemical moieties [20,21,22]. In fact, by traditional methods based on functionalized silanes, chemically modified surfaces have been provided for subsequent grafting of large biological molecules in a strong and homogeneous way [23,24,25,26].

In the last decade, bioactive mesoporous materials have been experimentally and/or clinically studied as scaffolds for cell attachment, proliferation, and differentiation on account of their uniform pore size distribution, high specific surface area and tunable pore size [27,28,29,30,31,32].

In our recent previous work, we disclosed intriguing possibilities for the use of an alternative silica porous materials from diatoms as a natural, easily available, multifunctional material for regenerative medicine applications [33]. In our model system, straightforward chemical manipulation endows *Thalassosiria weissflogii* silica shells with multiple properties: (1) loading and delivery of ciprofloxacin antibiotic useful for treatment of infections associated with orthopedic or dental devices; (2) reactive oxygen species (ROS) scavenging function potentially able to prevent inflammatory adverse side effects; and (3) bone cell adhesion and proliferation.

It is important to emphasize that in all these strategies, surface chemistry plays a central role in designing efficient inorganic-organic mesostructured platforms for bionanotechnological applications: starting from drug synthesis, continuing with the chemical modification of the surface for a better specificity and a longer lifetime in the action medium and ending with the modulation or tuning the functions and properties of the scaffolds with the aim of adapting them to the disease status [22,34,35,36].

Along with the application of diatom-based mesoporous materials for bone tissue engineering, special attention should be given to the possibility of covalently grafting biomolecules or osteoinductive agents (peptides, proteins and growth factors) to the surface of the 3D scaffolds, which would act as “attractive” signals for bone cells and promote the bone regeneration process. In this context, the effect of the chemical functionalization of the diatom surfaces on bone cell adhesion and proliferation deserves to be investigated.

Here, we present results on chemical modifications of diatom surfaces with organosilanes agents (3-mercaptopropyl-trimethoxysilane (MPTMS) and 3-aminopropyl-triethoxysilane (APTES)) leading to amino-coated and mercapto-coated biosilica microcapsules. After a full chemical and physical characterization (X-ray photoelectron spectroscopy (XPS), fourier transform infrared (FTIR) and scanning electron microscopy (SEM) analyses), normal human dermal fibroblasts (NHDF) and human osteosarcoma Saos-2 cell line (Saos-2) adhesion and growth on bare and silane-coated diatom frustules were investigated.

## 2. Materials and Methods

### 2.1. Diatom Culture

The pelagic centric diatom *Thalassiosira weissflogii* (culture collection of algae and protozoa, CCAP strain 1085/10) was grown in a sterile f/2-enriched seawater medium [37]. The resulting salinity of the seawater medium was about 3.8%–3.875%. In the first 4 days of subculture, glucose was added (0.55 mg·L^−1^) for enhancing cell viability, and sodium sulfate (4.26 g·L^−1^) for increasing photosynthesis yields, as reported in the literature [38,39]. The medium was enriched with Na_2_SiO_3_·9H_2_O, trace metals, and a vitamin mix and a final pH 8.0 was achieved [40]. Moreover, in order to avoid bacterial contamination, a low amount of kanamycin (0.5 mg·L^−1^) was added. Growth was controlled at 18–20 °C under a continuous photon flux density (PFD) provided by cool-white fluorescent tubes. The light source was placed 15 cm away from cultures. The light/dark cycle was 12 h illumination/12 h darkness and minimal air change (basal oxygen influx) was guaranteed by ventilation through sterile filters applied onto tubes. Cell density and cell parameters were measured using standard counting in a Burker hemocytometer every 4 days of growth, monitored for 14 days, as previously reported [41].

### 2.2. Biosilica Cleaning Procedures

After 14 days of growth, 20 mL of Thalassiosira w. cells dispersion (10^6^ cells/mL) were collected by centrifugation (1250 rpm, 12′) and the pellet was rinsed with bidistilled water. Organic matter was removed by acid treatment with aqueous H_2_SO_4_ (300 µL, 98% *w*/*w*) and oxidation with KMnO_4_ (1 mg) at 80 °C for 30′. A further oxidation step with hydrogen peroxide (600 µL) at 90 °C for 4 h was performed to activate the oxydrilic groups onto SiO_2_ surfaces. This treatment was followed by several washing steps and centrifugations (1350 rpm, 12′). The resulting white pellet was dried under vacuum at room temperature (2 h).

### 2.3. Grafting on Biosilica with 3-Aminopropyl-trimethoxysilane (APTES)

Dry and cleaned frustules (15 mg) were stirred in 450 µL of toluene in a vial for 10′ at room temperature under dry nitrogen atmosphere, followed by an addition of 5 μL of bidistilled water to the mixture. After 1–2 h of stirring, 9 μL of APTES were added (3 μL every 10′) and refluxed at 70 °C for 1 h. The modified particles were collected by centrifugation (1200 rpm for 15′), filtered and washed thoroughly with toluene (3×), isopropanol (3×) and water (3×). A rinse of the collected biosilica (16 mg) in 5% triethylamine/ethanol was performed to remove any physiosorbed APTES molecules, followed by a drying in a vacuum desiccator at ambient conditions. A final suspension in isopropanol/water (1:1) allowed us to obtain a good-quality functionalized biosilica dispersion [42].

### 2.4. Grafting on Biosilica with 3-Mercaptopropyl-trimethoxysilane (MPTMS)

Dry and cleaned frustules (15 mg) were added to 450 μL of dry toluene and stirred for 10′ at room temperature under dry nitrogen atmosphere. An amount of 5 μL of bidistilled water was added to the mixture and stirred at room temperature. After 1 h, 15 μL of MPTMS were added dropwise to avoid MPTMS di-polymerization and refluxed at 60 °C for 6 h. The modified particles were collected by centrifugation (1200 rpm for 15′), filtered and washed thoroughly with toluene (3×), isopropanol (3×) and water (3×) followed by drying in a vacuum desiccator at ambient conditions (2 h) [25].

### 2.5. SEM Analyses of Bare and Functionalized Frustules

SEM analysis was carried out by means of a field emission gun (FEG) Zeiss SUPRA 40 apparatus at a voltage of 3 KV at tilting angle of 0°. Dried samples before and after functionalization were resuspended in 200 μL of methanol to obtain a low-density frustule suspension. The preparation of samples for SEM characterization was performed using this simple protocol: (a) 1 cm × 1 cm silicon wafers were washed with acetone (3×), with ethanol (3×) and with isopropanol (3×), with 10 s of sonication (80 W) per washing step; (b) 5 μL of frustule suspension were drop casted on a hot plate (60 °C) 5 times; (c) thermal annealing and solvent evaporation (70 °C for 1 h) was performed.

### 2.6. Compositional Analysis (XPS)

XPS was performed by a Thermo VG Theta Probe spectrometer equipped with a micro-spot monochromatized Al Kα source (hν 1486.6 eV; 300 W; 300 µm spot size); base pressure 1 × 10^−10^ mbar. Photoelectrons were collected at a take-off angle of 53°, for a sampling depth of ~10 nm. Differential or non-uniform charging of the samples was correct with an Ar-fed flood gun (Mod. 822-06 FG; 400 µA emission current, 40 V extraction voltage; operating pressure 2 × 10^−7^ mbar). Low and high resolution spectra were acquired with pass energy of 150 eV and 100 eV, respectively. Bare, APTES and MPTMS-frustules were casted on a platinum substrate, which prevents differential charging surface phenomena resulting in alteration in the photoelectron peak shape and FWHM, and a disentangling of the contribution of the casted powder from the underlying substrate. The surface chemical composition (in at %) of each sample is determined by evaluating the integrated peak areas of the photoelectron peaks and the respective sensitivity factors, as described elsewhere. Data processing was performed using the “AVANTAGE” software. The spectra were calibrated at C1s peak post acquisition, at 285.0 eV binding energy, for the aliphatic hydrocarbon C–C/C–H bonds. The C1s, O1s, N1s Si2p and S2p peaks were best fitted by making a first trial without constraints, and then fitting with an imposed number of components by using the constraint that all components of a peak have the same full width at half maximum (FWHM: 1.3 eV).

### 2.7. Biosilica Layers Preparation for Cell Growth

Cell culture experiments were performed on modified and native frustules deposited on a round, 13 mm diameter, glass cover slide. Glass samples were activated by piranha solution to create exposed hydroxyl groups for further depositions. Glass slides were treated with a solution of 2:1 H_2_O_2_ (30% *v*/*v*)/H_2_SO_4_ conc. (98%), at 85 °C for 2 h. Then three washing steps in bidistilled water, in acetone, in ethanol and in 1:1 water ethanol were performed. One set of glass slides (G) were used as controls for cell growth. Bare-frustules (F), APTES-frustules (F-NH_2_) and MPTMS-frustules (F-SH) were deposited via three steps of drop-casting onto another three sets of slides. After drop-casting of 10 µL of each suspension on slides, we performed 10′ of pre-annealing (to dry solvents) at 50 °C on a hot plate, before checking an almost uniform coverage and frustule density via SEM and microscopy analyses (Supplmentary Materials, Figures S1 and S2). The three sets of frustule samples were annealed at 60 °C for 2 h under nitrogen (in a glass Petri dish) with the aim of obtaining frustule layers resistant to a medium culture contact and a soft scraping.

### 2.8. Cell-Culture Experiments

Both human Saos-2 osteoblastic cell line (ICLC) and NHDF fibroblasts (PromoCell) were used for cell culture experiments. Cells were routinely grown in Dulbecco’s modified Eagle’s medium (DMEM) (Sigma Chemical Co., St. Louis, MO, USA) supplemented with 10% heat-inactivated fetal bovine serum (FBS), 50 IU/mL penicillin, 50 IU/mL streptomycin, and 200 mM glutamine and maintained at 37 °C in a saturated humid atmosphere containing 95% air and 5% CO_2_ in 75 cm^2^ tissue culture-treated flasks (Barloworld Scientific, Stone, UK). For cell culture experiments, cells were detached with a trypsin/EDTA solution (Sigma) and resuspended in the correct medium at a concentration of 1 × 10^5^ cells/mL, and 1 mL was seeded on samples placed in 24-well plates, and grown for 24 and 96 h.

### 2.9. Viability and Proliferation Assay

To measure the metabolic activity of the Saos-2 and NHDF cells, which allow us to analyze cell viability and growth, MTT (3-(4,5-dimethylthiazol-2-yl)-2,5-diphenyltetrazolium bromide reactant) analysis was performed according to the manufacturer’s instructions (Sigma, Milan, Italy). Briefly, the enzyme substrate, 3-(4,5-dimethylthiazol-2-yl)-2,5-diphenyl tetrazolium bromide, at a 5 mg/mL concentration in PBS, was added to the cells, at a final concentration of 1/10 in respect to the cell culture medium. Cells were incubated for about 2–3 h at 37 °C/95% CO_2_ until the substrate was converted to violet water-insoluble formazan crystals, and accumulated into the cytoplasm of viable cells from which it was solubilized using 1 mL of lysis buffer (500 mL isopropanol, 55 mL Triton X-100, 4.58 mL HCl). The absorbance of formazan produced was measured at 590 nm with a spectrophotometer.

### 2.10. Cell Morphological Analysis: Coomassie Blue Staining and Image Analysis

Saos-2 cells seeded on the substrata and analyzed at different cell culture times were fixed in 4% paraformaldehyde/PBS solution (15 min) and stained for 3 min in a dye solution (0.2% Coomassie Brilliant Blue R250 (Sigma), 50% methanol, 10% acetic acid). Cells adhering to different substrata were observed at different magnifications with a phase contrast microscope (Leica DM ILI, Leica Microsystems Srl, Milan, Italy); at least 15 digital images per sample were acquired through a CCD camera (Leica DC100, Leica Microsystems, Germany; Meyer Instruments, Houston, TX, USA) at different magnifications. Images acquired on different samples and at different times were analyzed with the Image J image analysis software (National Institute of Health, New York, NY, USA) to evaluate different parameters such as substratum area covered by cells and morphological parameters of single cells, perimeter and circularity upon varying the time.

### 2.11. Statistical Analysis

Statistical analyses were assessed by a two-way ANOVA test within groups, followed by a Bonferroni post-test, using the GraphPad Prism Software version 4.00 for Windows (GraphPad Software, San Diego, CA, USA). Differences were considered statistically significant for *p* < 0.01.

### 2.12. Cell Morphological Analysis: SEM

For SEM observations, cells grown on samples for the two different time lapses were fixed with 2.5% glutaraldehyde/0.1 M sodium cacodylate solution, post-fixed with a 1% osmium tetroxide 0.1 M sodium cacodylate solution, and dehydrated using a series of ethanol/water solutions (20%, 40%, 50%, 70%, 90% and 100%). Because the samples were nonconductive, they were coated with a thin layer of Au before SEM examination by using a plasma sputtering apparatus. A (FEG) Zeiss SUPRA 40 (Axiomat, Zeiss, Germany) operating at 20 kV was used to evaluate cell/frustule interaction and distribution on the entire sample surface.

### 2.13. Cell Morphological Analysis: Indirect Immunofluorescence and Cytoskeleton Observation

In order to observe the actin cytoskeleton, cells grown on samples for the two different time lapses were fixed in 4% formaldehyde/PBS solution at room temperature (RT) for 20 min, permealized with PBS containing 0.1% Triton X-100, and incubated with Alexa Fluor488 phalloidin (Molecular Probes, Eugene, OR, USA) at RT and for 20–30 min. After rinsing, samples were mounted in Vectashield fluorescent mountant with DAPI (Vector Laboratories, Peterborough, UK) and then observed by means of an epifluorescence microscope (Axiomat, Zeiss, Oberkochen, Germany).

### 2.14. Cell Morphological Analysis: Preparation of T-BTZ-T Annealed Frustule Substrates

In order to better visualize frustule layers under cell layers, after the deposition of frustules on glass substrates, frustule layers were stained with a solution of T-BTZ-T dye. The synthesis of the silanized version of the dye has been previously published [43]. For the staining solution preparation, 1 mg of yellow-red emitting T-BTZ-T (silanized version) was mixed in 1 mL of pure DMSO (dimethyl sulfoxide), and the final solution was filtered (0.2 µm Ø). The solution (100 µL) was casted on frustule-glass samples and a pre-thermal annealing (50 °C) was performed. Temperature was then brought to 90–100 °C for 30′ and the samples were washed three times in DMSO-EtOH (1:1) washing solutions, and then dried.

## 3. Results and Discussion

### 3.1. Chemical Functionalization of Diatom Frustules

**Figure 1:**
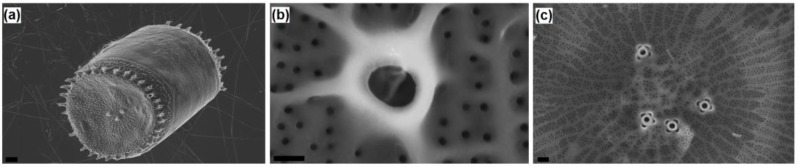
Scanning electron microscopy (SEM) images of a *Thalassiosira weissflogii* frustules: (**a**) after soft-acid treatment (1 h in HCl-MeOH) the whole structure is preserved; (**b**,**c**) after hard acidic oxidative treatment (4 h in H_2_SO_4_-H_2_O_2_), the intricate pattern of pores is clearly visible. Markers: (**a**) 1 µm; (**b**) 100 nm; (**c**) 200 nm.

The centric *Thalassosira weissflogii* diatom was grown as reported in the Materials and Methods section. After growth, the diatom protoplasm was removed from the cells by oxidative/acidic treatments affording pure discrete nanoporous microcapsules with an average size of 10–15 μm (see Figures S1 and S2 in Supplementary Materials). The morphology of these mesoporous biosilica materials was examined by SEM analyses showing distinct and separated diatom frustules without aggregations and the absence of detrimental effects onto the pore surface due to the cleaning treatments (Figure 1).

**Figure 2:**
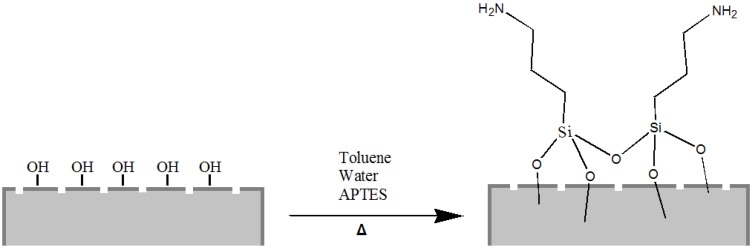
Scheme of diatom frustule passivation with 3-aminopropyl-trimethoxysilane (APTES).

Starting from our previous experience of chemical modification of biosilica from diatoms [33], we have covalently functionalized the silica shells with 3-aminopropyl-trimethoxysilane (APTES), a popular surface-modification agent widely used for a number of applications in promoting steady adsorption between organic compounds (proteins, drugs) and silica substrates (Figure 2).

**Figure 3:**
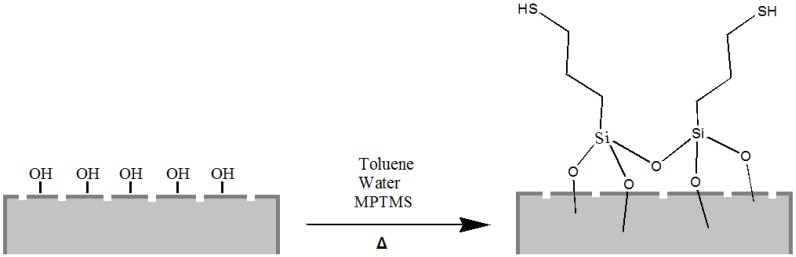
Scheme of diatom frustule passivation with 3-mercaptopropyl-trimethoxysilane (MPTMS).

Moreover, it is well known that thiol moieties have been incorporated into the diatom during frustule synthesis via co-condensation of organoalkoxysilanes within the SDV (silica deposition vesicle) of the parent diatom [44], whereas self-assembled monolayers of 3-mercaptopropyl-trimethoxysilane (MPTMS) have been used to chemically modify the surface of diatom silica microparticles for the adsorption of mercury ions Hg(II) [25]. Following this last reported procedure, we also carried out the grafting of organosilanes containing mercapto (–SH) groups on the frustule surface aiming to obtain mercapto-coated biosilica (Figure 3).

**Figure 4:**
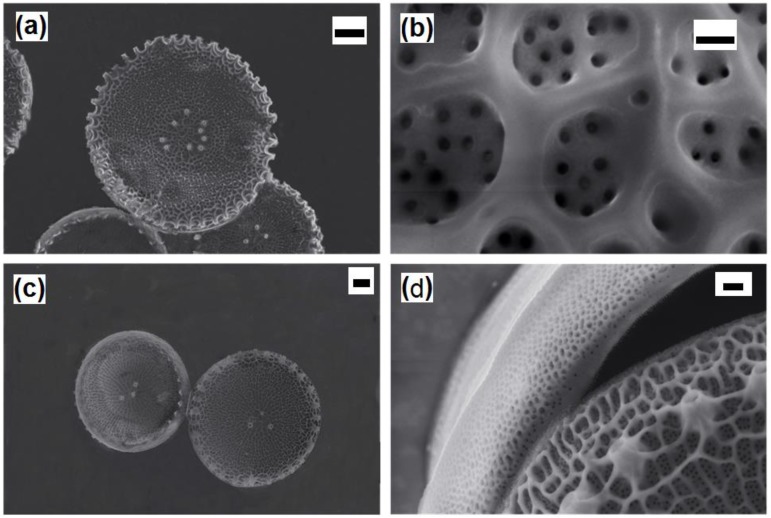
SEM images of (**a**) the whole frustule valves of *Thalassiosira weissflogii*; (**b**) the pore patterning after amino-silanization; frustule valves; (**c**) valves-girdle nanotexturing; (**d**) after mercapto-silanization. After both the chemical modification, the pores are not occluded. Markers: (**a**) 2 µm; (**b**) 100 nm; (**a**) 1 µm; (**d**) 200 nm.

After surface modifications, SEM analyses confirmed no topographical changes in functionalized diatom frustules (Figure 4).

### 3.2. Characterization of Modified Frustules by FTIR Spectroscopy

**Figure 5:**
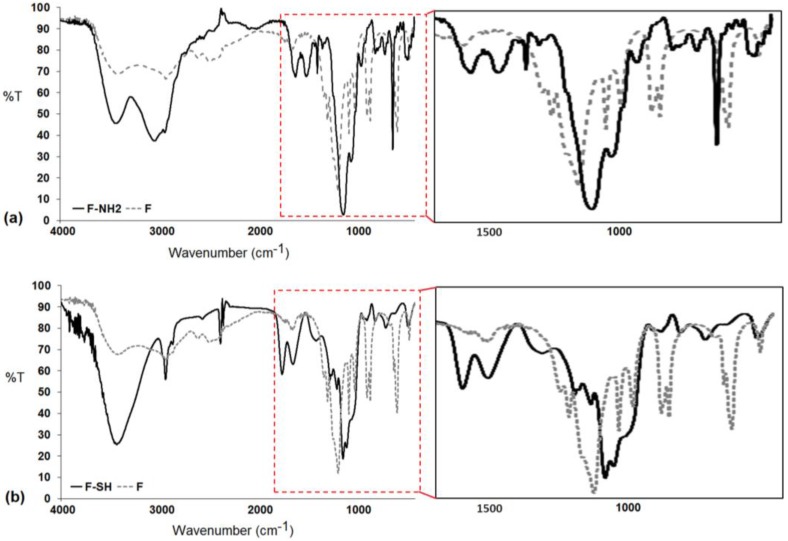
Fourier transform infrared (FTIR) spectra of (**a**) the diatom frustules before and after chemical functionalization with APTES molecules (in the right side, the region between 1800 cm^−1^ and 500 cm^−1^ is magnified); (**b**) the diatom frustules before and after chemical functionalization with MPTMS molecules (in the right side, the region between 1800 cm^−1^ and 500 cm^−1^ is magnified).

FTIR spectroscopy was used to investigate the mercapto- and aminosilane grafting on the frustule surface of the F-SH and the F-NH_2_ samples. In Figure 5, a comparison between the FTIR spectra before and after the amino silanization of the diatoms shows that, despite the extensive pretreatment, some organic moieties are still present on the surface of the bare frustules, as evidenced by the symmetric and asymmetric ν(CH_2_) observed at 2930 and 2840 cm^−1^, respectively. However, the most relevant wavenumber range is from 950 to 1250 cm^−1^ as it includes Si–O–Si vibrational modes. The characteristic signals at 1100 cm^−1^ and 1080 cm^−1^ corresponding to the stretching of Si–OR in the triethoxysilanes moyieties are replaced with two strong bands at 1040 and 1130 cm^−1^ corresponding to the Si–O–Si(biosilica) stretching. The NH_2_ scissor vibration found at 1570 cm^−1^ confirms the presence of the terminal groups of the APTES molecules on the biosilica surface. In addition to this mode, other features at 1490 and 1605 cm^−1^ correspond to the symmetric and asymmetric NH_3_^+^ deformation modes, indicative of amine group protonation probably due to the acid–base reaction of the silane NH_2_ groups with the –OH groups on the diatom surface [41,45].

The FTIR spectra comparison corresponding to the biosilica before and after the grafting of the MPTMS (Figure 5b) clearly shows the modification of the Si–O–Si stretching mode region after the mercapto-silanization (1088 and 1127 cm^−1^), the presence of the propyl chains of the MPTMS molecules for the CH_2_ asymmetric and symmetric stretching modes at 2930 and 2860 cm^−1^, respectively, and the presence of the –SH group for the stretching vibration around 2558 cm^−1^ [25].

### 3.3. XPS Data of Functionalized and Native Samples

**Figure 6:**
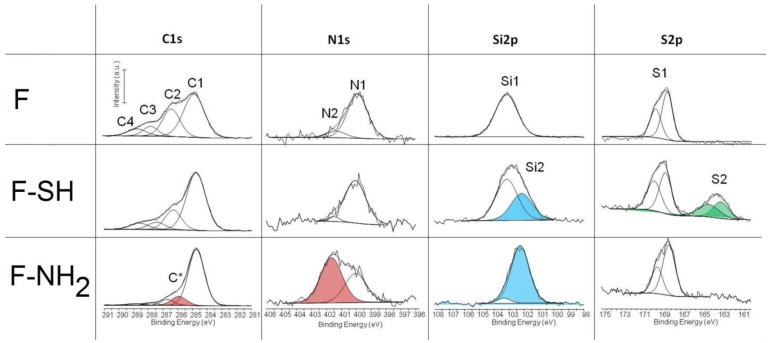
Best fitting of C1s, N1s, Si2p and S2p X-ray photoelectron spectroscopy (XPS) peaks.

The acquisition of C1s, N1s, Si2p and S2p high resolution spectra give some important information about the success of the functionalization whatever the process used. The results of deconvolution of all high resolution peaks acquired on different test groups are reported in Figure 6. The chemical composition of materials before and after surface functionalization are reported in Table S1 (Supplementary Materials). The peaks that confirm the efficacy of the immobilization are highlighted in red, green and light blue. The Cls peak is decomposed into four components with a full width at half maximum (FWHM) in the range of 1.2–1.3 eV: (C1) 284.8 eV as B.E. due to hydrocarbon; (C2) 286.5 eV B.E. due to alcohol; (C3) 287.9 eV due to hemiacetal, acetal, amide; (C4) 289.1 eV due to carboxyl (COOH) or ester functions (COOR). Slight changes are revealed in the case of F-SH in respect to F samples while a totally different C1s spectrum was acquired on F-NH_2_ samples. In this case, an additional component is present (C*) and centered at 285.8 eV, which is a characteristic carbon of amino groups. This result attests for a successful immobilization of APTES on frustules. These results were confirmed by the deconvolution of N1s spectra. The N1s peak was decomposed into two components: N1 component at 399.9 eV attributed to amide or amine functions and a component at 402.1 eV (N2) that is due to ammonium, protonated amine or quaternary ammonium. The N2 component (red peak) becomes predominant in the case of F-NH_2_ samples, thereby confirming once more the presence of protonated –NH_2_ on the frustule surface.

The Si2p peak of the F sample is characterized by a single peak centered at 103.2 eV as B.E. (Si1) which is typical of silica. For other samples, the variation of shape and position of the Si2p peak can be described by considering this component and a second component near 102.1 eV, which may be attributed to silicon covalently bound to organic moieties (silicic ester). This component is much more relevant in the case of F-SH samples probably due to a more homogeneous deposition of frustules. Note that each component was considered as a symmetric peak. It was not found relevant to consider that it is in fact a doublet with the 2p3/2 and 2p1/2 contributions separated by 0.6 eV. Finally, the S2p peak was deconvoluted into two doublets, the first one centered at 169 eV as B.E. that is characteristic of sulfate and the other one centered at 163.5 eV that is characteristic of sulfur covalently bound to organic moieties. Each doublet corresponds to the 2p3/2 and 2p1/2 contributions separated by 1.2 eV each other.

### 3.4. Cell Viability Assay on Modified Diatom’s Biosilica

In recent works, surface modifications of biointerfaces (silica nanoparticles, nanotubes, nanotextured surfaces) are reported to influence cell viabilities for non-adherent and adherent cells [46,47,48,49]. Osteogenic activity on amine-rich substrates like polydopaminated-coated Ti implants and on mercapto-functionalized surfaces, has been demonstrated to interact directly with cell proteins controlling cell adhesion and proliferation [50]. Also, TiO_2_ nanotubes (ATNs) chemically modified with 3-aminopropyltrimethoxysilane (APTMS) and 3-mercaptopropyltrimethoxysilane (MPTMS) have been tested for the growth of NIH/3T3 fibroblasts [51]. In our previous study, nanostructured biosilica produced by *Thalassiosira weissflogii* diatoms were covalently functionalized leading to an antioxidant TEMPO-biosilica which was demonstrated to be a suitable material for fibroblasts and osteoblast-like cell growth [33].

**Figure 7:**
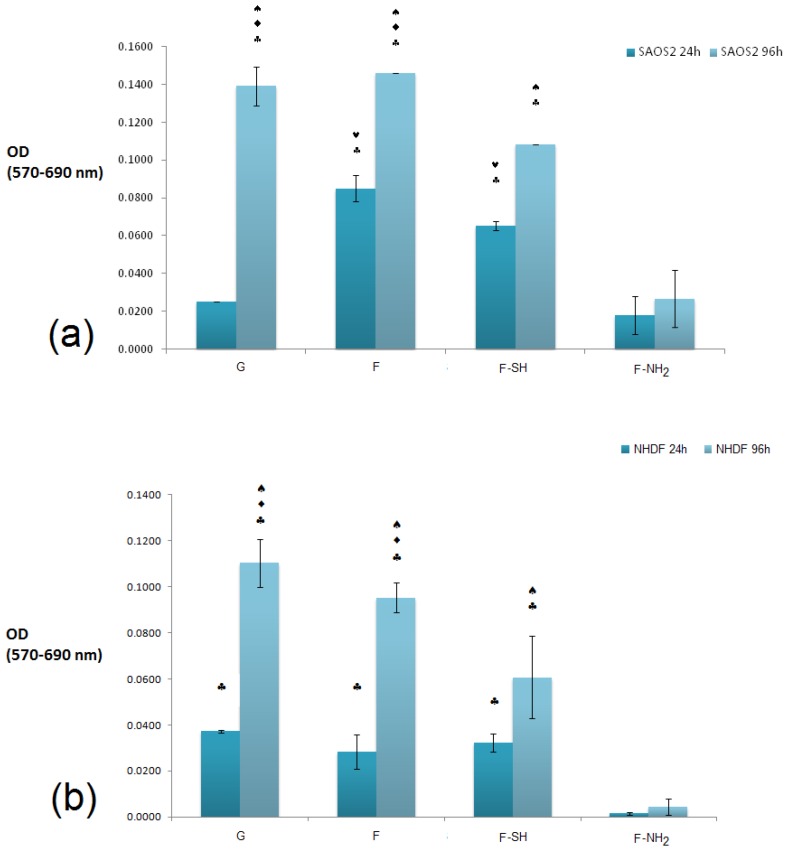
A comparison of viability values through optical densitis (OD, 570 nm–690 nm for background correction) of Saos-2. (**a**) normal human dermal fibroblasts (NHDF) (**b**) cell growth recorded at 24 and 96 h. The cells were grown on glass (G), functionalized (F-SH and F-NH_2_) and native frustules (F) deposed on glass. Statistical differences among means were calculated by a two-way ANOVA test followed by a Bonferroni’s post-test: ♠ *p* < 0.01 vs. 24h; ♥ *p* < 0.01 vs. G; ♦ *p* < 0.01 vs. F-SH; ♣ *p* < 0.01 vs. F-NH_2_; ✜ *p* < 0.01 vs. F.

The cytocompatibility of bare and chemically modified (with APTES and MPTMS) frustule samples were tested by studying cell viability, spreading and proliferation of both NHDF fibroblasts and Saos-2 osteoblasts cells [52,53,54]. We choose these two cell lines because they have been generally used for testing biocompatibility of silicate-bioceramics co-composites [55]. The NHDF and Saos-2 cells are normally exploited to investigate the adhesion and proliferation of silica-derived scaffold and bioglass [56,57]. Saos-2 cells are also an osteoblast model for testing biosilica-based materials and their co-composites [58,59]. Results of the MTT viability tests of both NHDF and Saos-2 cell lines carried out on F, F-NH_2_ and F-SH samples (uniform covering glass slides, see Figures S3 and S4 in Supplementary Materials) are shown in Figure 7.

It is evident that a statistically significant cell growth (96 h vs. 24 h, *p* < 0.01) is present for all substrates except for F-NH_2_. This may indicate that both cell types were not affected either by the meso-structure or by the SH group, but were negatively affected when exposed to –NH_2_ groups.

When the viability levels among the different substrates are compared for either 24 or 96 h, a difference in behavior is dependent on cell type, especially at the 24 h time point. In fact, viability levels recorded from the Saos-2 line (Figure 7a) revealed an interesting pattern of behavior: after 24 h, cell viability was higher on the F and F-SH substrata than on G control (*p* < 0.05). A higher affinity towards the mesoporosity of the frustules than the G control (glass) could be the reason why Saos-2 reaches high levels of cell viability just after 24 h. When the MTT values for NHDF cells were examined (Figure 7b), these differences among structured and flat samples were not found even after the 24 h time point. Again, the presence of –NH_2_ groups on frustules was toxic even for Saos-2, but the results suggest that the toxicity of the F-NH_2_ is weakly balanced by the presence of mesostructure, which positively affects Saos-2 viability.

Toxicity due to the –NH_2_ functional group for biomaterials which have to be employed in scaffold production for bone tissue regeneration is not so surprising. Very recently, a problem of cytotoxicity related to nanoparticle-cell interfaces has been investigated [60]. In particular, cationic silica nanoparticles (mainly from amino groups) have been demonstrated to produce plasma−membrane integrity problems, stronger mitochondrial and lysosomal damage, and a higher number of autophagosomes than anionic nanoparticles (for example, from sulfonic and carboxylic groups). This negative affection seems to occur even before internalization of the nanoparticles caused by the cell surface−cationic nanoparticle interaction at physiological pH.

Moreover, from a recent and specific study on correlation between cell viabilities and surface functionalization of silanized magnesium alloy, the amino group has been again reported to negatively promote cell adhesion and proliferation of Saos-2 cells, likely due to a supposed massive albumin subtraction from cell media [61].

Promotion and enhancement of osteosarcoma cell adhesion and proliferation has been demonstrated to be favorable when the surfaces are functionalized with hydrophobic groups, while less favorable with polar groups, especially with the amine moiety [62].

Lastly, toxicities to the use of amino-functionalized mesoporous silica surfaces for biomedical purposes have been correlated with intracellular ROS formation in cell lines [63].

### 3.5. Morphological Analysis of Cell Behavior

The differences observed for viability tests do not give any information about morphology and behavior of cells grown on native and functionalized frustules. In order to analyze if cell morphology was also dependent on surface chemical composition of frustules, cells were dyed with a Coomassie Blue solution, and observed at the optical microscope at different magnifications.

**Figure 8:**
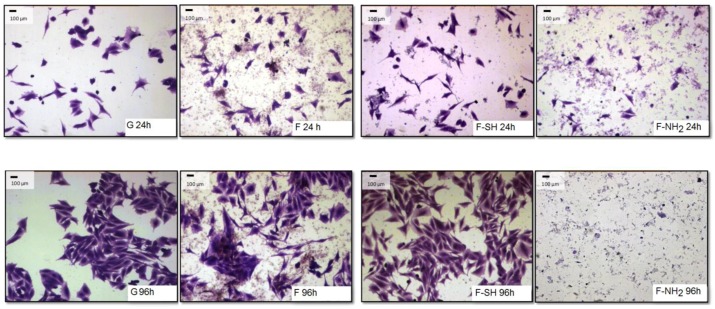
Paraformaldehyde (PFA) fixed and Coomassie Blue-stained Saos-2 cells grown for 24 and 96 h on the different substrates.

**Figure 9:**
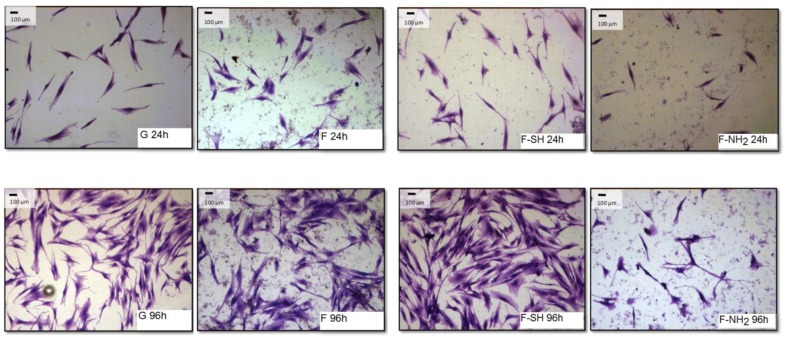
Paraformaldehyde (PFA) fixed and Coomassie Blue-stained NHDF cells grown for 24 and 96 h on the different substrates.

As shown in Figure 8 and Figure 9, after 96 h of growth on G, bare F and F-SH substrates, both cell types formed clusters. The normal shape of cells is maintained per cell type: both cell lines were more rounded at 24 h, while after 96 h Saos-2 cells were spread in all directions and NHDF extruded as rod-like structures. This was not the case for Saos-2 and NHDF cells grown on F-NH_2_ samples, where only distinct cells were present after 96 h of growth and any clusterization was absent. Again, morphological investigations underlined a cell suffering in the presence of –NH_2_ groups, while cells on the other two samples showed good cell behavior (in terms of spreading and coverage during growth) (see Supplementary Materials for high-resolution Coomassie of cells grown on bare diatom’s biosilica, Figure S5).

In order to obtain a quantitative analysis of cell morphology grown on different substrates, acquired images were subjected to analysis through the Image J software. A first type of analysis was performed on low-magnification images, like the ones shown in Figure 8 and Figure 9, and aimed at calculating the percentage of a substrate’s area covered by cells.

**Figure 10:**
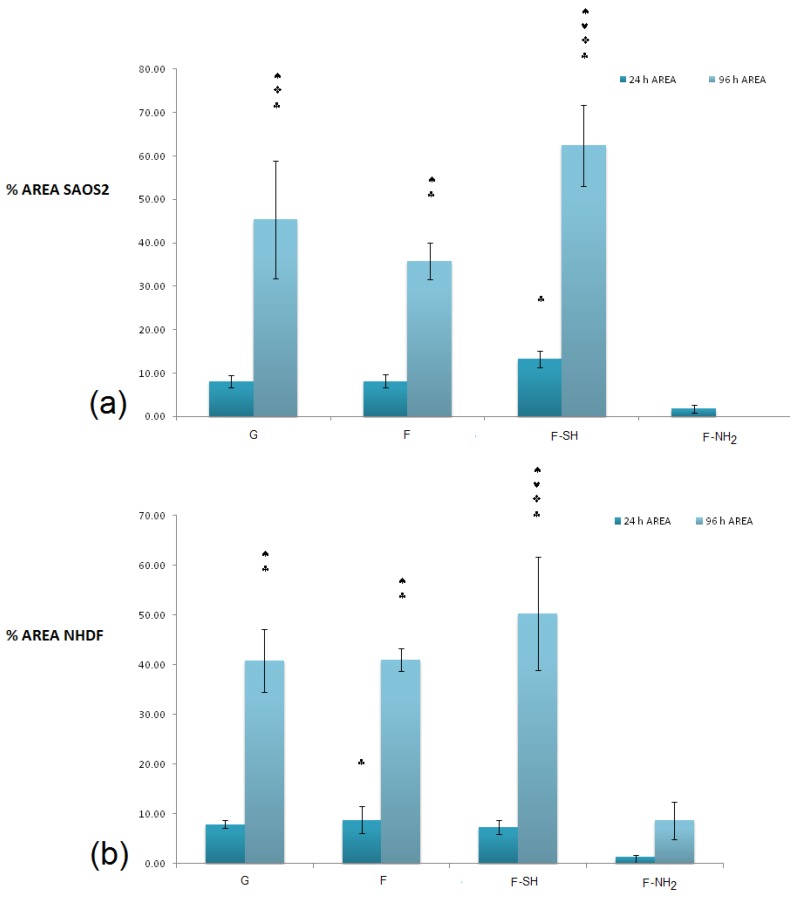
The % of substrate area covered by Saos-2 (**a**) and NHDF cells (**b**) grown on the different substrates after 24 and 96 h. Statistical differences among means were calculated by a two-way ANOVA test followed by a Bonferroni’s post-test: ♠ *p* < 0.01 vs. 24 h; ♥ *p* < 0.01 vs. G; ♦ *p* < 0.01 vs. F-SH; ♣ *p* < 0.01 vs. F-NH_2_; ✜ *p* < 0.01 vs. F.

**Figure 11:**
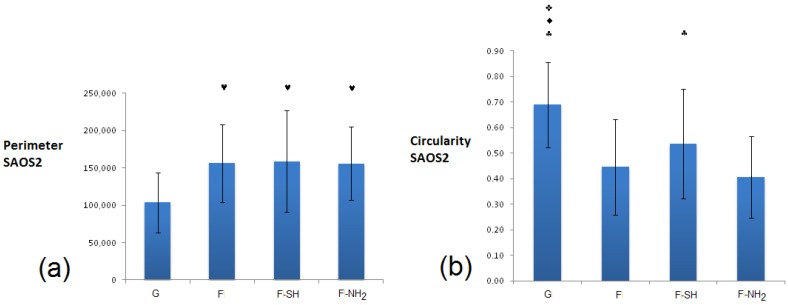
Shape factors of Saos-2: perimeter (in pixels) (**a**) and circularity (arbitrary units) (**b**) grown for 24 h on glass and frustules containing substrates. Statistical differences among means were calculated by a two-way ANOVA test followed by a Bonferroni’s post-test: ♠ *p* < 0.01 vs. 24 h; ♥ *p* < 0.01 vs. G; ♦ *p* < 0.01 vs. F-SH; ♣ *p* < 0.01 vs. F-NH_2_; ✜ *p* < 0.01 vs. F.

**Figure 12:**
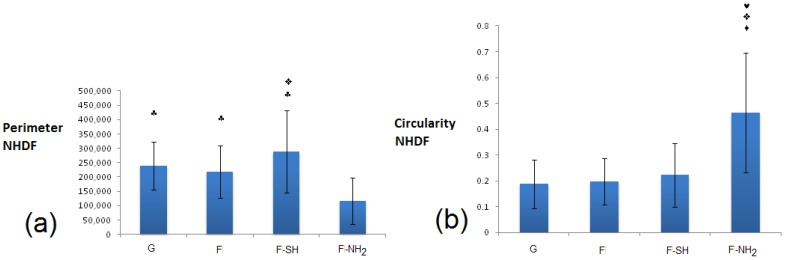
Shape factors of NHDF: perimeter (in pixels) (**a**) and circularity (arbitrary units) (**b**) grown for 24 h on glass and frustules containing substrates. Statistical differences among means were calculated by a two-way ANOVA test followed by a Bonferroni’s post-test: ♠ *p* < 0.01 vs. 24 h; ♥ *p* < 0.01 vs. G; ♦ *p* < 0.01 vs. F-SH; ♣ *p* < 0.01 vs. F-NH_2_; ✜ *p* < 0.01 vs. F.

In terms of coverage of substrate area (Figure 10), there were no differences between the various substrates for the value of the area covered (with the exception of the –NH_2_ frustules) after 24 h, while at 96 h an increased coverage on the mercapto-functionalized substrates was observed. This discrepancy between MTT results and substrate area’s cell coverage can be due to differences in the cells’ morphology. To verify this hypothesis, an analysis of single-cell morphology can be helpful. In particular, perimeter and circularity of single cells adhering to different samples were calculated. Good scaffold-like materials let cell perimeter reach high values but kept circularity at low values [64,65]. This study was performed only at 24 h since after 96 h of growth most of the cells formed clusters and a non-sufficient number of single cells, required for statistical validity, were present. Shape factors (perimeter and circularity averages) were calculated by recording the values of at least 60 cells of that population (Figure 11 and Figure 12).

In the case of Saos-2 (Figure 11), cells grown on all frustule-coated substrates (F, F-SH and F-NH_2_) exhibited de facto higher perimeter and lower circularity values with respect to glass, and there are no significant differences among all biosilica-based materials. These results suggest a higher degree of Saos 2 interaction with the biosilica substrates, maybe due to the acquisition of a more elongated and starry morphology.

For NHDF cells grown on glass and biosilica-based substrates (Figure 12), the situation must be deeply evaluated. Circularity at 24 h was similarly low in all substrates (G, F, F-SH), while perimeter factors were similarly high in the same previous substrates (G, F, F-SH). For the F-NH_2_, however, the situation is the opposite because NHDF on F-NH_2_ exhibited high circularity and low perimeter. This data suggests that cells on amino-functionalized frustules tend to be more circular, less spread and with no pyramidal morphology, likely underlining the absence of membrane specializations. Cells grown on G, F, F-SH possess indicative shapes of proper fibroblast maturation.

### 3.6. Interaction of Cell Filopodia and Lamellipodia with Mesostructures

With the aim of deeply investigating frustule-cell interactions, high magnification images of cells deposited on layers of frustules were performed by fluorescence and SEM microscopy. Both types of cells interact with frustules by long filopodia and large lamellipodia. Lamellipodia and filopodia are cell protrusions consisting in a network of, respectively, short, branched and long parallel actin filaments. They therefore play fundamental roles in cell migration.

**Figure 13:**
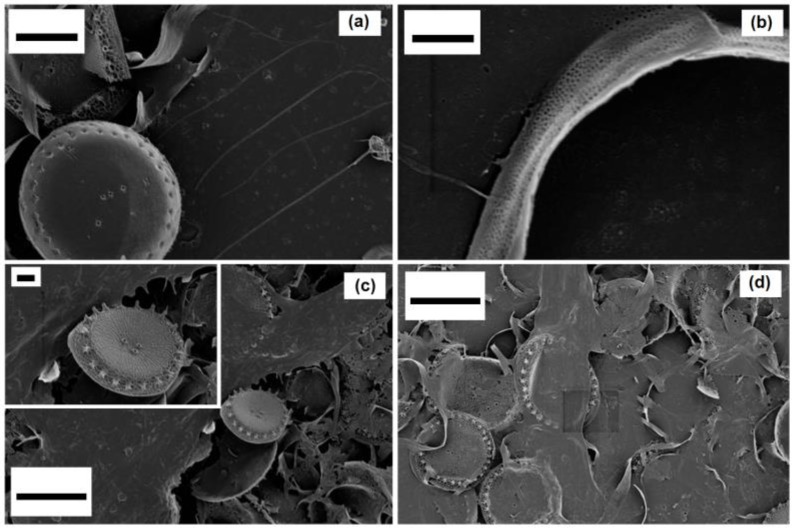
SEM pictures of NHDF (**a**–**c**) and Saos-2 (**d**) cells grown for 24 h on frustules containing surfaces. (**a**) Cell filopodia directing toward a frustule; (**b**) cell filopodium interacting with a girdle; (**c**) cell’s lamellipodia interacting with a frustule (inset at higher magnification); (**d**) cells covering structured surface. Marker: (**a**) 5 µm; (**b**) 1 µm; 10 µm for (**c**) and (**d**) (2 µm for inset).

SEM images in Figure 13 clearly show how NHDF and Saos-2 cell covers adhere and proliferate on frustules deposited on glass, intimately connecting the nanostructured valves and girdles by both filopodia and lamellopodia.

Although multicellular organisms contain a wide array of actin filament assemblies, the behavior of lamellipodial and filopodial bundles and the contractile actin stress fibers in the cytoplasm reveals how the actin cytoskeleton modifies itself when cells interact with the surface of the substrata. Cell migration, changes in cells’ shape, and adhesive properties are affected by the morphology of the substrata on which the cell growth occurs, phenomena caused by a continuous remodeling of the actin cytoskeleton.

In order to investigate how the frustules affect the modelling of actin cytoskeleton, a phalloidin staining of cells has been performed.

The study of modelling of actin cytoskeleton, the nuclear organization and cell−biosilica interactions were performed in a unique triple staining of Saos-2 cytoskeleton (via phalloidin staining), nuclei (via DAPI) and frustules (via staining with T-BTZ-T) [43]. In particular, this triple staining was performed to improve visualization of biosilica under cell monolayers. The T-BTZ-T dye is yellow-red emitting when visualized with FITC filters, so samples treated with a triple staining combination (F-T-BTZ-T samples, (c) and (d)) appear more clear.

**Figure 14:**
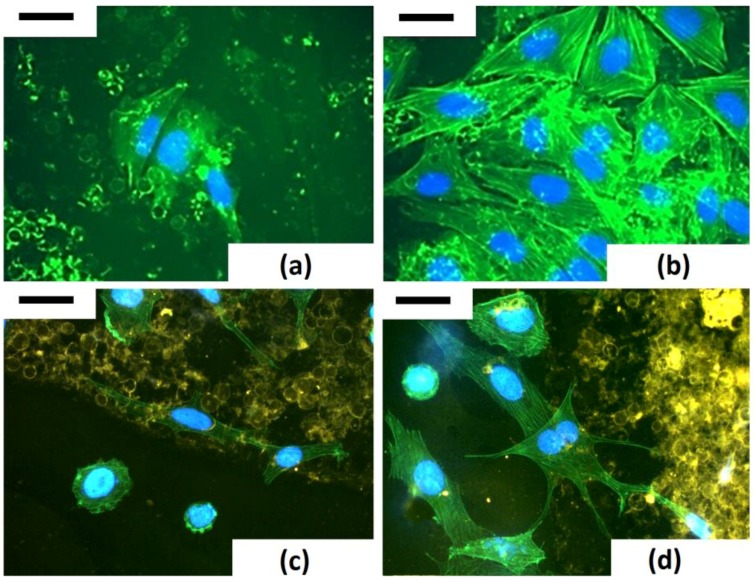
Saos-2 morphological investigations via fluorescence microscopy after double (DAPI + atto-falloidin 488, F 24 (**a**) and F 96 h (**b**)) and triple staining with T-BTZ-T (T-BTZ-T + DAPI + atto-falloidin 488, F-T-BTZ-T 24 (**c**) and 96 h (**d**)). Marker: 50 µm.

These images confirm that the substrate affects morphology and, specifically, cells grown on such material are stimulated to emit long ramified prolongations and to extend connections to the substrate. Bare frustules are already exploitable due to their weak auto-fluorescence (see Figure 14, (a) F 24 h sample), but the green auto-fluorescence is not easily recognizable when cells are denser ((b) F 96 h sample). Figure 14 clearly shows that cells interacting with frustules exhibit well-developed stress fibers while cells adhering on “empty” surfaces are rounded, with a low level of actin polymerization and perinuclear dots of actin.

## 4. Conclusions

In this study, we investigated the chemical modification of the diatom’s frustule surface with 3-aminopropyl-trimethoxysilane (APTES) and with 3-mercaptopropyl-trimethoxysilane (MPTMS), and performed adhesion/proliferation experiments of bone cells such as NHDF and Saos-2 cells on APTES and MPTMS-modified diatom frustules.

We compared cell viabilities, shape factors, percentage of cell−substratum adhesion and morphological analysis of Saos-2 type, and NHDF cells grown on bare and functionalized biosilica-coated glass. We primarily confirmed that the two cell lines positively reacted to the mesostructure of bare biosilica shells, showing higher vitality and sometimes better shape factors than the internal positive control (glass). Cell adhesion/proliferation on functionalized biosilica-coated substrata encountered serious difficulties when the mesostructure of biosilica is associated with amino group functionalities. Conversely, the mercapto- group has exceptionally improved vitality and shape factors of the two cells lines with respect to bare biosilica. Comparing the two cell lines’ behavior, Saos-2 exhibited more sensitivity to the mesostructure compared to NHDF, in terms of viability, while the NHDF were more sensitive to the functionalization in terms of viability and also shape factors. We finally deeply investigated cell−single-frustule interaction, showing that cells use proper membrane specialization (lamellipodia, filopodia) when approaching biosilica substructures (crowns, girdles, valves), and they reach maximal stretching during the adhesion process onto biosilica substrates, taking pyramidal, irregular, stretched shapes.

However, the major challenge for these materials, which are foreign to the body, is to achieve satisfactory tissue integration and differentiation. Natural biomaterials such as biosilica from diatoms may overcome this challenge because they are biocompatible.

## Supplementary Materials

The following are available online at www.mdpi.com/ 2306-5354/3/4/35/s1, Figure S1: SEM images of a *Thalassiosira weissflogii* frustule (a) after soft-acid treatment, the whole structure is preserved (1 h in HCl-MetOH); (b,c) after hard-acid-oxidative treatment, the intricate pattern of pores is clearly visible. Markers: (a) 1 µm; (b) 100 nm; (c) 200 nm. On the right: Measurements of average size of valves, obtained through acid-oxidative (strong) treatment of living diatoms after 1–4 days of cultures and 14 days of cultures; number of nanopores in girdle units (total area A of 500 × 500 nm), Figure S2: SEM images of the whole frustule valves of *Thalassiosira weissflogii* (a) and of the pore patterning (b) after amino-silanization; frustule valves (c) and valves-girdle nanotexturing (d) after mercapto-silanization. After both the chemical modification, the pores are not occluded. Markers: (a) 2 µm; (b) 100 nm; (c) 1 µm; (d) 200 nm. On the right: Measurements of average size of functionalized valves, after acid-oxidative (strong) treatment of living diatoms from 14-day-old cultures; number of nanopores in girdle units (total area A of 500 × 500 nm), Figure S3: SEM images of frustule-coated-slides for cell culture experiments: F-NH_2_ (a) and F-SH (b). Marker: 10 µm, 200 nm and 10 µm, Figure S4: Optical microscopy of 13 cm glass coverslip layered with (a) non-modified frustules (b) mercapto-modified and (c) amino-modified frustules, Figure S5: High magnification pictures of NHDF (a) and Saos-2 (b) cells interacting with frustules. Marker: 100 µm, Table S1: Chemical composition determined by XPS of the native (F) and chemically modified (F-NH_2_ and F-SH) samples referred to silicon content.
