# Supplementary material for: Biosilica from Living Diatoms: Investigations on Biocompatibility of Bare and Chemically Modified Thalassiosira weissflogii Silica Shells
Source: Bioengineering (Basel). 2016 Dec 16;3(4):35. doi: 10.3390/bioengineering3040035 (PMC5597278; doi:10.3390/bioengineering3040035)
Supplement: Supplementary file 1 [file bioengineering-03-00035-s001.pdf]

bioengineering

Supplementary Materials: Biosilica from Living Diatoms: Investigations on Biocompatibility of Bare and Chemically Modified *Thalassiosira weissflogii* Silica Shells

Stefania Roberta Cicco, Danilo Vona, Roberto Gristina, Eloisa Sardella, Roberta Ragni, Marco Lo Presti and Gianluca Maria Farinola

1. Scanning Electron Microscopy (SEM) Analyses

The architecture parameters of biosilica (the pore and particle morphologies, [65]) of the *Thalassiosira weissflogii* frustules were examined by SEM images showing (Figures S1 and S2) distinct and separated diatom frustules without aggregations and the absence of detrimental effects of the cleaning treatments.

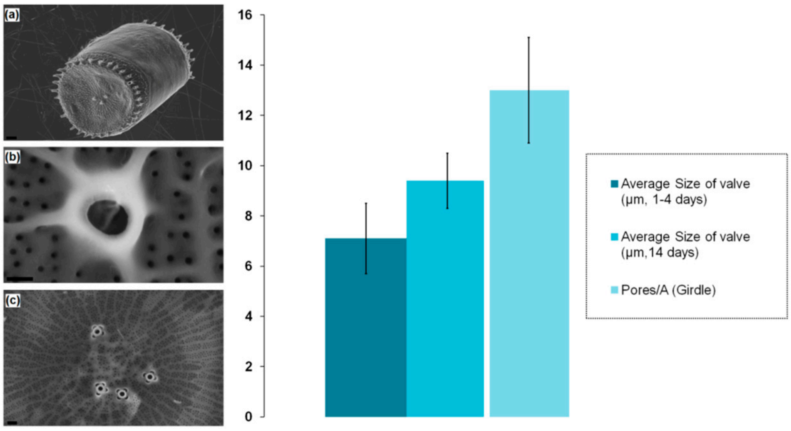

Figure S1. SEM images of a *Thalassiosira weissflogii* frustule (**a**) after soft-acid treatment, the whole structure is preserved (1 h in HCl-MetOH); (**b**,**c**) after hard-acid-oxidative treatment, the intricate pattern of pores is clearly visible. Markers: (**a**) 1 μm; (**b**) 100 nm; (**c**) 200 nm. On the right: Measurements of average size of valves, obtained through acid-oxidative (strong) treatment of living diatoms after 1–4 days of cultures and 14 days of cultures; number of nanopores in girdle units (total area A of 500 × 500 nm).

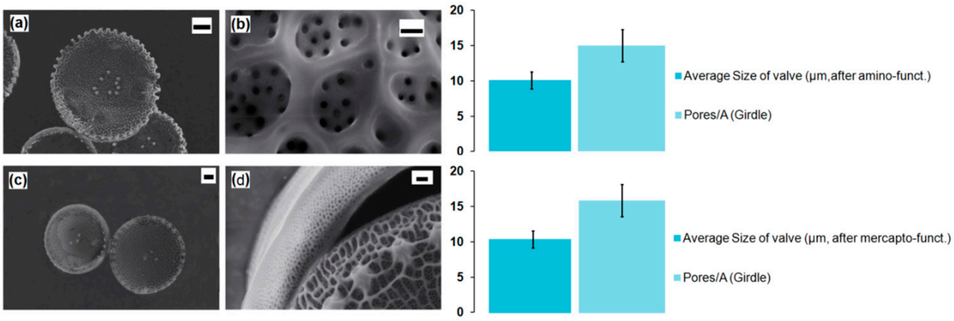

Figure S2. SEM images of the whole frustule valves of *Thalassiosira weissflogii* (**a**) and of the pore patterning (**b**) after amino-silanization; frustule valves (**c**) and valves-girdle nanotexturing (**d**) after mercapto-silanization. After both the chemical modification, the pores are not occluded. Markers: (**a**) 2 μm; (**b**) 100 nm; (**c**) 1 μm; (**d**) 200 nm. On the right: Measurements of average size of functionalized valves, after acid-oxidative (strong) treatment of living diatoms from 14-day-old cultures; number of nanopores in girdle units (total area A of 500 × 500 nm).

2. X-Ray Photoelectron Spectroscopy (XPS) Analyses

The chemical composition of materials before and after surface functionalization are reported in Table S1. Due to a probable absence of homogeneity of the sample through all the explored thicknesses or the presence of endogenous S- and N-derived contaminants coming from living organic moieties (in bare samples, F), only slight differences were observed in terms of chemical composition among all test groups. It is worth mentioning that XPS of complex materials like micro and nanostructured ones, as in the case of frustules, is affected by several factors. Above all is the geometry of such systems and, subsequently, sometimes—as in this case—the comparison between chemical composition of differently functionalized surfaces is not enough to address some conclusions. The highest C/Si ratio reported for F-NH_2 samples should be the result of a more homogeneous coverage of the substrate with amino silane molecules than F-SH.

Table S1. Chemical composition determined by XPS of the native (F) and chemically modified (F-NH_2 and F-SH) samples referred to silicon content.

Samples	C/Si	O/Si	K/Si	S/Si	Mg/Si	Na/Si	Cl/Si
**F**	2.4 ± 0.1	5.0 ± 0.10	0.25 ± 0.15	0.66 ± 0.13	0.19 ± 0.20	0.21 ± 0.30	0.09 ± 0.23
**F-NH_2**	16.0 ± 0.1	4.8 ± 0.22	0	0.73 ± 0.15	0	0.02 ± 0.13	0
**F-SH**	3.39 ± 0.5	2.6 ± 0.06	0	0.4 ± 0.23	0	0.13 ± 0.20	0.09 ± 0.12

3. SEM Images of Frustule-Coated Slides for Cell Culture Experiments

The SEM images (Figures S3 and S4) show an overlapping of several (rigid) valves and (flexible, banana peel) girdles leading to a quasi three-dimensional stratum of nanostructured biosilica. The analyses with the optical microscopy of the frustule-coated coverslips demonstrate a uniform coverage (89% ± 3% of the available area) for both the samples. For this reason, the differences eventually observed in cell growth can be solely attributed to the chemical modification of the biosilica surface.

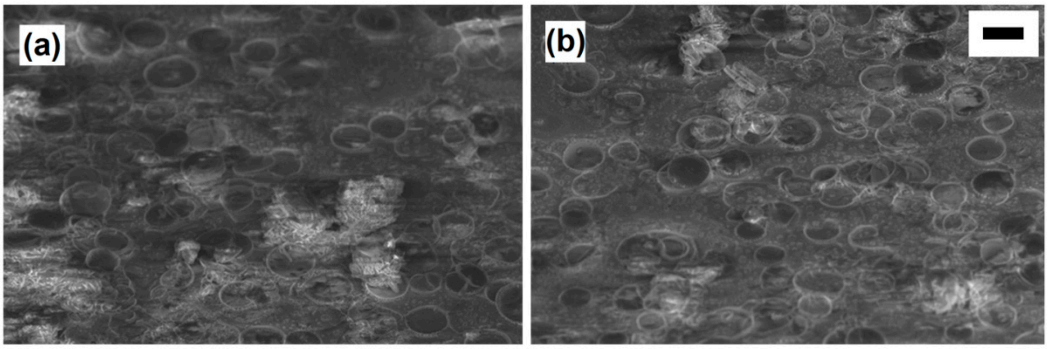

Figure S3. SEM images of frustule-coated-slides for cell culture experiments: F-NH_2 (**a**) and F-SH (**b**). Marker: 10 μm, 200 nm and 10 μm.

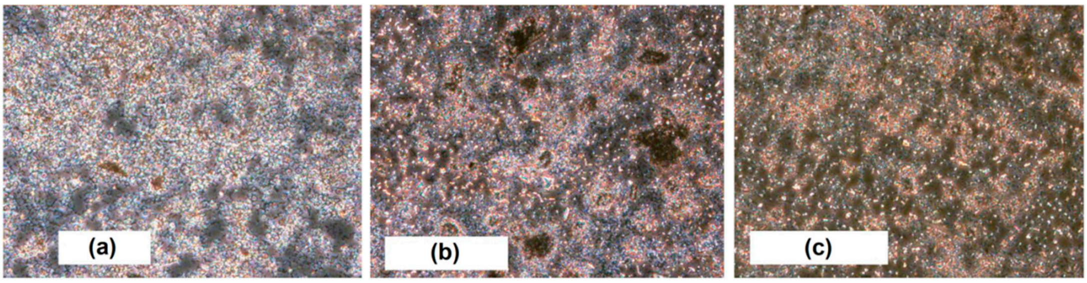

Figure S4. Optical microscopy of 13 cm glass coverslip layered with (**a**) non-modified frustules (**b**) mercapto-modified and (**c**) amino-modified frustules.

4. High-Resolution Coomassie of Cells Grown on Bare Diatom's Biosilica

Images in Figure S5 show the maximal spreading of NHDF cells (a) and Saos-2 cells (b) on frustules punctually deposed near large non-deposed area, in order to appreciate differences.

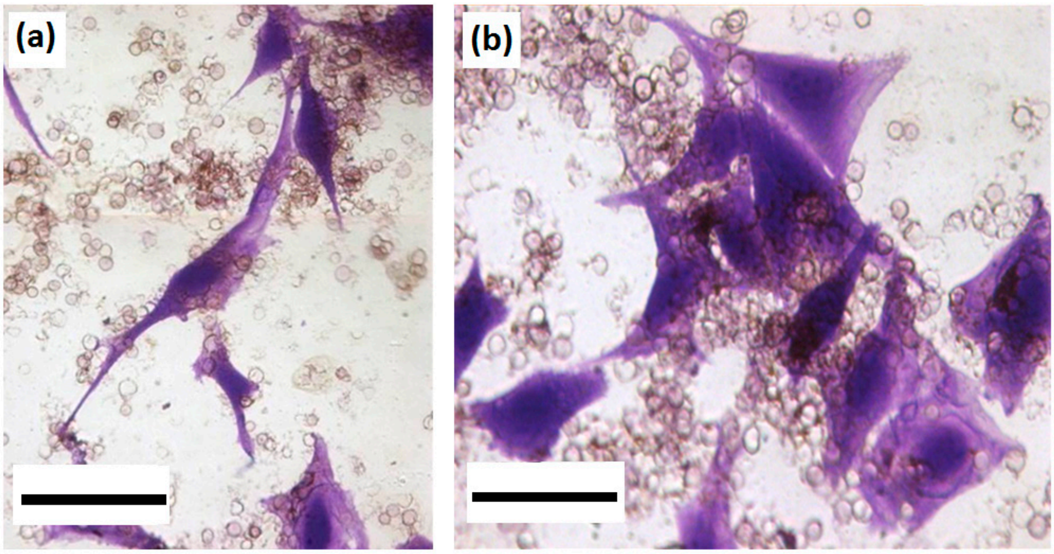

Figure S5. High magnification pictures of NHDF (**a**) and Saos-2 (**b**) cells interacting with frustules. Marker: 100 μm.

© 2016 by the authors. Submitted for possible open access publication under the terms and conditions of the Creative Commons Attribution (CC-BY) license (http://creativecommons.org/licenses/by/4.0/).
